# Microstructural Alterations in Asymptomatic and Symptomatic Patients with Spinocerebellar Ataxia Type 3: A Tract-Based Spatial Statistics Study

**DOI:** 10.3389/fneur.2017.00714

**Published:** 2017-12-22

**Authors:** Xinwei Wu, Xinxin Liao, Yafeng Zhan, Cheng Cheng, Wei Shen, Mufang Huang, Zhifan Zhou, Zheng Wang, Zilong Qiu, Wu Xing, Weihua Liao, Beisha Tang, Lu Shen

**Affiliations:** ^1^Department of Neurology, Xiangya Hospital, Central South University, Changsha, China; ^2^Institute of Neuroscience, CAS Center for Excellence in Brain Science and Intelligence Technology, Chinese Academy of Sciences, Shanghai, China; ^3^Department of Radiology, Xiangya Hospital, Central South University, Changsha, China; ^4^Key Laboratory of Hunan Province in Neurodegenerative Disorders, Central South University, Changsha, China; ^5^State Key Laboratory of Medical Genetics, Changsha, China; ^6^National Clinical Research Center for Geriatric Disease, Changsha, China; ^7^Parkinson’s Disease Center of Beijing Institute for Brain Disorders, Beijing, China; ^8^Collaboration Innovation Center for Brain Science, Shanghai, China; ^9^Collaboration Innovation Center for Genetics and Development, Changsha, China

**Keywords:** spinocerebellar ataxia type 3, asymptomatic, tract-based spatial statistics, white matter tracts, ataxia

## Abstract

**Objective:**

Spinocerebellar ataxia type 3 (SCA3) is the most commonly occurring type of autosomal dominant spinocerebellar ataxia. The present study aims to investigate progressive changes in white matter (WM) fiber in asymptomatic and symptomatic patients with SCA3.

**Methods:**

A total of 62 participants were included in this study. Among them, 16 were asymptomatic mutation carriers (pre-SCA3), 22 were SCA3 patients with clinical symptoms, and 24 were normal controls (NC). Group comparison of tract-based spatial statistics was performed to identify microstructural abnormalities at different SCA3 disease stages.

**Results:**

Decreased fractional anisotropy (FA) and increased mean diffusivity (MD) were found in the left inferior cerebellar peduncle and superior cerebellar peduncle (SCP) in the pre-SCA3 group compared with NC. The symptomatic SCA3 group showed brain-wide WM tracts impairment in both supratentorial and infratentorial networks, and the mean FA value of the WM skeleton showed a significantly negative correlation with the International Cooperative Ataxia Rating Scale (ICARS) scores. Specifically, FA of the bilateral posterior limb of the internal capsule negatively correlated with SCA3 disease duration. We also found that FA values in the right medial lemniscus and SCP negatively correlated with ICARS scores, whereas FA in the right posterior thalamic radiation positively correlated with Montreal Cognitive Assessment scores. In addition, MD in the middle cerebellar peduncle, left anterior limb of internal capsule, external capsule, and superior corona radiate positively correlated with ICARS scores in SCA3 patients.

**Conclusion:**

WM microstructural changes are present even in the asymptomatic stages of SCA3. In individuals in which the disease has progressed to the symptomatic stage, the integrity of WM fibers across the whole brain is affected. Furthermore, abnormalities in WM tracts are closely related to SCA3 disease severity, including movement disorder and cognitive dysfunction. These findings can deepen our understanding of the neural basis of SCA3 dysfunction.

## Introduction

Spinocerebellar ataxia type 3 (SCA3) is the most globally prevalent form of autosomal dominant spinocerebellar ataxia (1). It is characterized by cerebellar ataxia, pyramidal and extrapyramidal signs, peripheral neuropathy, ophthalmoparesis, and cognitive dysfunction (2). Evidence has revealed that CAG repeat expansion in the gene encoding the Ataxin-3 protein results in SCA3 disease (3). Studies have indicated that there is a long period of gradual accrual of pathological changes before the occurrence of SCA3 symptoms (4). Fortunately, because it is a monogenetic disease, early detection of SCA3 through genetic testing is possible. Since most asymptomatic mutation carriers have few or no clinical symptoms, it is necessary to find biomarkers either in body fluids or through imaging changes during the asymptomatic stage of SCA3, to enable assessment of disease progression and prediction of therapeutic outcomes.

So far, neuropathological studies have shown widespread involvement of the central nervous system, including the cerebellum, pons, spinal cord, as well as the cerebral cortex and basal ganglia, in SCA3 (4, 5). Neuroimaging studies have also revealed alterations in brain structure and function in SCA3 patients. Specifically, previous studies have showed generalized reduction in brain volume, with significant gray matter (GM) atrophy in the pons and the vermis (6), as well as in supratentorial regions including the frontal lobe, temporal lobe, parietal lobe, occipital lobe, putamen, and caudate (7–10). White matter (WM) atrophy has also been found in the lateral thalamus, brainstem, and cerebellum (6). Moreover, diffusion-weighted imaging (DWI), which is capable of revealing microstructural characteristics of the human brain by quantifying the integrity of WM tracts *in vivo* through measurement of water diffusion directionality (11), has revealed microstructural changes of the WM fibers in SCA3. However, these studies have mainly focused on WM damage in infratentorial structures such as the cerebellum and brainstem. Recent DWI studies have demonstrated that microstructural damage of WM is widespread in SCA3 (2, 12). However, little is known about abnormalities in WM in the asymptomatic stage of SCA3.

Tract-based spatial statistics (TBSS), using non-linear image transformation, is a technique based on DWI. It is a technique that combines both voxel-wise and tractography-based analyses (13). TBSS increases the sensitivity and reliability of analysis of multi-subject diffusion imaging (14). It has been utilized for detecting alterations in subjects with movement or neurodegenerative disorders, such as Alzheimer’s dementia, Parkinson’s disease, and mild cognitive impairment (15–17).

In the present study, we applied TBSS analysis to investigate WM damage in asymptomatic and symptomatic SCA3 subjects and attempted to characterize the correlations between image findings and SCA3 clinical symptoms. We hypothesized that impaired WM fibers were present in asymptomatic and symptomatic SCA3 patients, and that damage of WM was associated with disease severity of SCA3.

## Materials and Methods

### Subjects

We recruited 16 asymptomatic mutation carriers (5 men, mean age 28.81 ± 7.19 years) and 22 genetically diagnosed SCA3 patients with clinical symptoms (14 men, mean age 43.36 ± 5.89 years) from the outpatient department of Xiangya Hospital, Central South University, between July 2012 and January 2016. The mean disease duration of the SCA3 group was 6.95 ± 4.36 years (range 1–20). Twenty-four healthy volunteers were included as a control group. None of the healthy participants had neurological diseases, psychiatric disorders, systemic metabolic diseases, or tumor. Age- and gender-matched subgroups of controls were selected for direct comparison with individual patient groups. All subjects gave their written informed consent prior to study participation. The privacy rights of participants were always observed. All procedures performed in studies involving human participants were approved by the Ethics Committee of Xiangya Hospital, Central South University in China, which was in accordance with the ethical standards of the institutional and/or national research committee, and with the 1964 Helsinki declaration and its later amendments or comparable ethical standards.

### Clinical Assessment

Spinocerebellar ataxia type 3 patients were interviewed to obtain information regarding age of onset, duration of disease, and family history. The International Cooperative Ataxia Rating Scale (ICARS) and Scale for the Assessment and Rating of Ataxia (SARA) were used to assess the severity of ataxia. The ICARS is a 0–100 score semiquantitative scale with 100 corresponding to maximal clinical deficits. It includes 19 items with 4 subscales: posture and gait disturbance, kinetic function, speech disorder, oculomotor disorders. The SARA is an 8-item performance-based scale, yielding a total score of 0 (no ataxia) to 40 (most severe ataxia). The asymptomatic mutation carriers had normal values on the SARA (≤1.5 points) (18). The Montreal Cognitive Assessment (MoCA) (19) was used to assess cognitive function.

### MRI Scanning

Brain MR imaging was performed on a 3.0 T MR scanner (Signa HDX, General Electric Healthcare, Milwaukee, WI, USA) at Xiangya Hospital Imaging Center. For all MR procedures, the head was immobilized using self-expanding foam cushions. Volumetric (3D) T1-weighted images were acquired: with thickness/gap = 1.5/0.5 mm, echo time (TE) = 3 ms, repetition time (TR) = 7.8 ms, TI = 380 ms, flip angle = 7°, matrix = 256 × 256, field of view (FOV) = 250 mm × 250 mm, voxel size = 0.5 mm × 0.5 mm × 0.5 mm. DTI data of all the subjects were obtained *via* a 35-direction non-collinear echo-planar sequence with the following parameters: thickness/gap = 3 mm/0, TE = 76 ms, TR = 6,000 ms, FA = 90°, matrix = 192 × 192, FOV = 240 mm × 240 mm, *b*-value = 1,000.

### Image Processing

The DTI DICOM data were first converted to the 4D Neuroimaging Informatics Technology Initiative format using MRIcron (http://people.cas.sc.edu/rorden/mricron/dcm2nii.html). The converted DTI data were then preprocessed using the FMRIB software library (20, 21). Specifically, distortions induced by eddy current were first corrected using EDDY tool. The eddy correction simultaneously corrected subject motion. B0 volumes of each participant were extracted using the Brain Extraction Tool and used as brain mask in native space. The preprocessed and eddy current corrected data was used to estimate the quantitative metric fractional anisotropy (FA). TBSS analysis was then performed on all FA data (22). First, we identified a common registration target and aligned all subject’s FA images to the target in standard Montreal Neurological Institute space by non-linear registrations (23); second, we converted all aligned FA images into 1 mm × 1 mm × 1 mm by affine registrations; the mean of all aligned FA images and its skeleton for all subjects were created, and each subject’s FA image was projected onto the skeleton. Using the same non-linear registration to the mean diffusivity (MD) data, all subject’s wrapped MD data were projected onto the original mean FA skeleton (24). Finally, voxel-wise statistical analyses across subjects on the common skeleton were performed (22, 25).

### Statistical Analysis

Two sample *t*-test was performed on the FA skeleton data to identify the abnormal WM fiber between pre-SCA3/SCA3 and control group by random tool. We also used the threshold-free cluster enhancement option in random to avoid the need for the arbitrary initial cluster-forming threshold. The significant statistical level was set at *p* < 0.05. Furthermore, the average MD of the abnormal WM identified in the FA analysis was extracted. Two sample *t*-test was performed on the average MD data between patient and control group. In addition, we analyzed the relationship between FA/MD of WM tract of JHU DTI-based white-matter atlases (26) and the clinical variables in the SCA3 groups using SPSS 19.0. The significant level was set at *p* < 0.05 for exploratory analysis.

## Results

### Demographics of Participants and Clinical Assessment

There were no significant differences in age and sex between the control group and the pre-SCA3 or SCA3 group (Table S1 in Supplementary Material). No clinically relevant differences were found in ICARS, SARA, and MoCA scores between the pre-SCA3 and control group. SCA3 patients showed significantly lower MOCA (*p* < 0.01), and higher SARA (*p* < 0.01) and ICARS scores (*p* < 0.01) compared with normal controls (NC).

### TBSS Analysis between the Cross-sectional Groups

**Figure 1:**
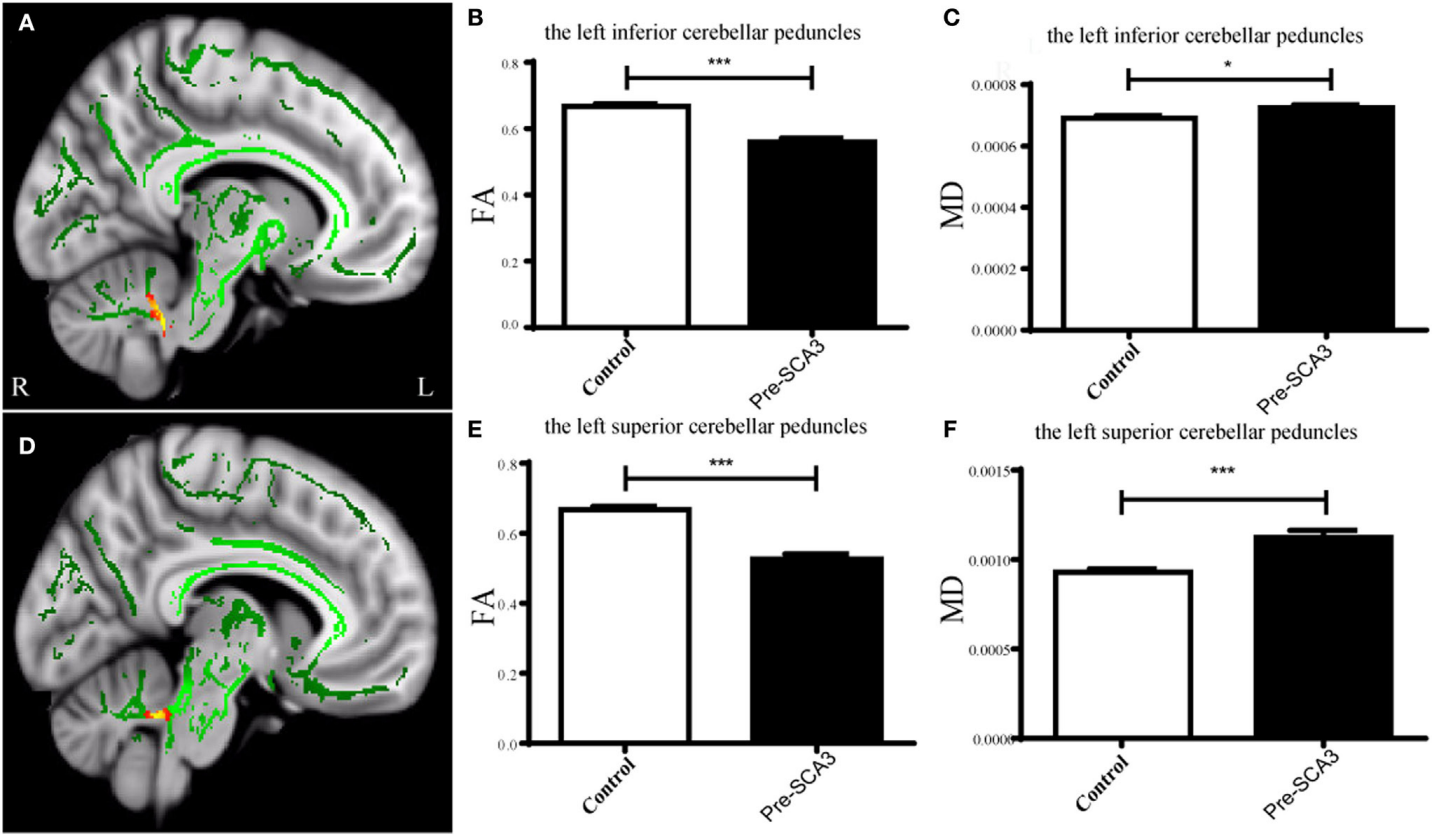
Tract-based spatial statistics analysis of the FA in pre-spinocerebellar ataxia type 3 (SCA3) patients compared with healthy controls. Pre-SCA3 patients showed decreased FA in the left inferior cerebellar peduncle **(A,B)** and left superior cerebellar peduncle **(D,E)**, whereas the mean diffusivity (MD) both increased **(C,F)** (green represents mean FA skeleton, red–yellow represents to set the display range to 0.95:1, *p* < 0.05, corrected).

**Figure 2:**
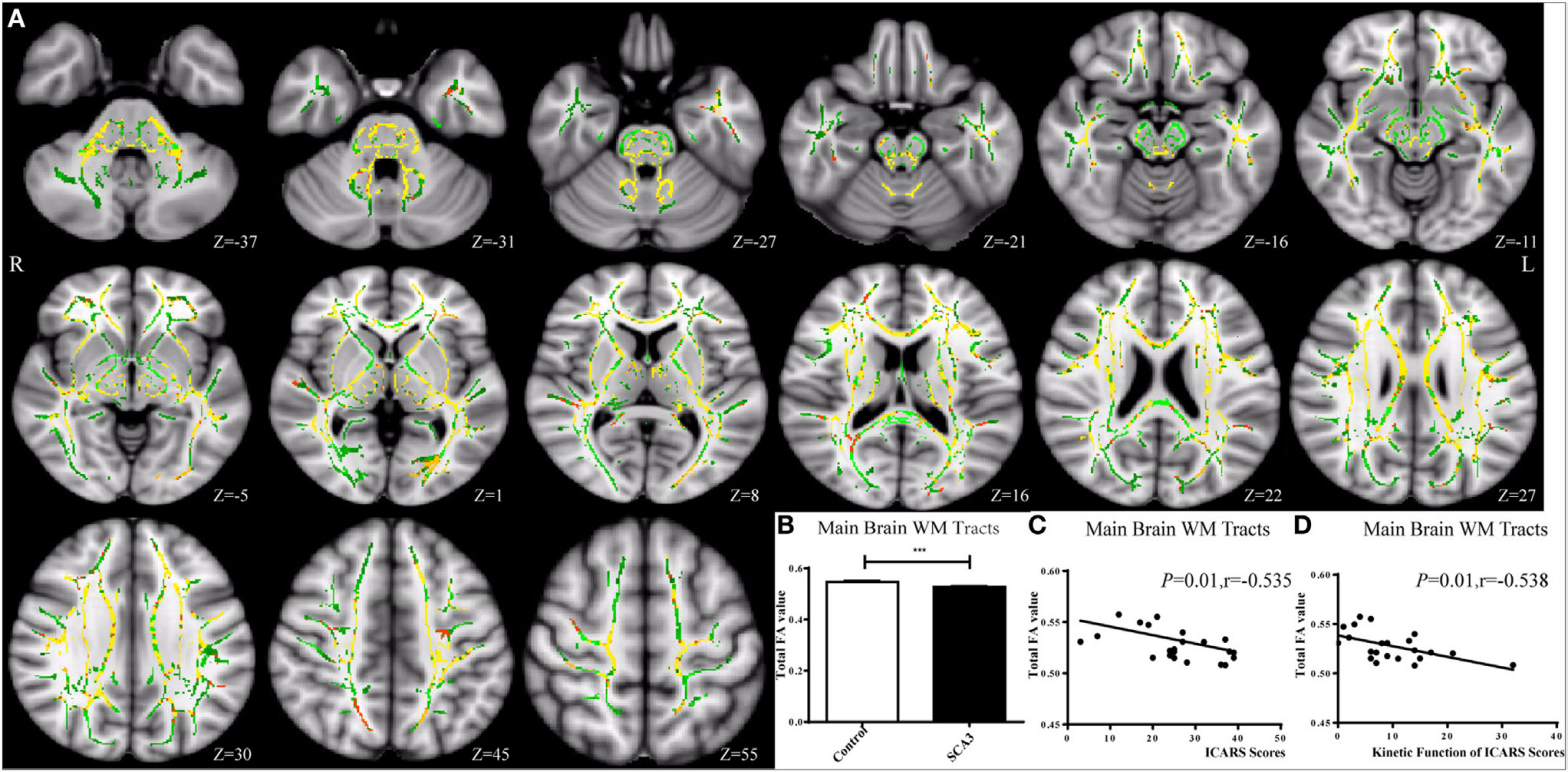
Tract-based spatial statistics analysis of the FA in spinocerebellar ataxia type 3 (SCA3) patients compared with healthy controls. SCA3 group showed decreased FA value of the mean white matter (WM) skeleton in the whole brain **(A,B)** (green represents mean FA skeleton, red–yellow represents to set the display range to 0.95:1, *p* < 0.05, corrected). A negative correlation was found between the total FA value of the mean WM skeleton and the total International Cooperative Ataxia Rating Scale (ICARS) scores **(C)** and its subscale of kinetic function **(D)** in SCA3 patients.

Tract-Based Spatial Statistics analysis revealed significant differences in FA and MD between the pre-SCA3 and NC in the left inferior cerebellar peduncles (ICP) and left superior cerebellar peduncles (SCP) (Figure 1; Table 1). Meanwhile, the SCA3 group showed widespread abnormal WM tracts in the whole brain (including the widespread supratentorial and infratentorial structures) and significantly decreased mean FA of the WM skeleton compared with NCs (Figures 2A,B). We further examined the WM tracts associated with the cerebellum and brainstem. We found that FA values of WM tracts in the cerebellum and brainstem, including the middle cerebellar peduncle (MCP), bilateral SCP, ICP, cerebral peduncle, pontine crossing tract, and medial lemniscus, were significantly decreased in SCA3 participants (Table 2). MD increases were also found both in the supratentorial and infratentorial regions including the MCP, SCP, ICP, cerebral peduncle, medial lemniscus, body and splenium of corpus callosum, superior corona radiate, anterior and posterior limb of internal capsule, and other WM tracts (Table 3).

**Table 1:** Decreased FA values and increased mean diffusivity (MD) in the left inferior cerebellar peduncle (ICP) and SCP of pre-spinocerebellar ataxia type 3 (SCA3) patients.

	FA (pre-SCA3)	FA (NC)	*p*-Value	MD (pre-SCA3) (×10^**−**3^)	MD (NC) (×10^**−**3^)	*p*-Value
Left ICP	0.56 ± 0.04	0.68 ± 0.04	<0.0001	0.72 ± 0.04	0.69 ± 0.04	<0.05
Left superior cerebellar peduncle	0.53 ± 0.06	0.67 ± 0.04	<0.0001	1.12 ± 0.16	0.92 ± 0.08	<0.001

**Table 2:** FA values of white matter tracts significantly decreased in cerebellum and brainstem in spinocerebellar ataxia type 3 (SCA3).

	FA value (SCA3)	FA value (NC)	*p*-Value
Middle cerebellar peduncle	0.63 ± 0.03	0.68 ± 0.02	<0.0001
Pontine crossing tract	0.56 ± 0.05	0.61 ± 0.03	<0.0001
Medial lemniscus			
R	0.64 ± 0.03	0.70 ± 0.03	<0.0001
L	0.64 ± 0.04	0.70 ± 0.03	<0.001
Inferior cerebellar peduncle			
R	0.47 ± 0.03	0.59 ± 0.02	<0.0001
L	0.47 ± 0.04	0.60 ± 0.03	<0.0001
Superior cerebellar peduncle			
R	0.58 ± 0.03	0.70 ± 0.03	<0.0001
L	0.61 ± 0.04	0.73 ± 0.03	<0.0001
Cerebral peduncle
R	0.74 ± 0.02	0.77 ± 0.02	<0.001
L	0.61 ± 0.03	0.64 ± 0.03	<0.001

**Table 3:** Mean diffusivity (MD) of white matter tracts significantly increased in spinocerebellar ataxia type 3 (SCA3).

	MD (SCA3) (×10^**−**3^)	MD(NC) (×10^**−**3^)	*p*-Value
Middle cerebellar peduncle	0.67 ± 0.04	0.61 ± 0.03	<0.0001
Inferior cerebellar peduncle			
R	0.69 ± 0.05	0.66 ± 0.03	<0.05
L	0.71 ± 0.04	0.66 ± 0.03	0.001
Superior cerebellar peduncle			
R	0.92 ± 0.07	0.78 ± 0.04	<0.0001
L	0.89 ± 0.06	0.76 ± 0.03	<0.0001
Medial lemniscus			
L	0.69 ± 0.06	0.66 ± 0.03	<0.05
Cerebral peduncle			
R	0.79 ± 0.04	0.75 ± 0.03	<0.05
L	0.74 ± 0.04	0.70 ± 0.03	<0.01
Body of corpus callosum	0.86 ± 0.03	0.83 ± 0.03	<0.01
Splenium of corpus callosum	0.69 ± 0.03	0.66 ± 0.02	<0.01
Anterior limb of internal capsule			
R	0.76 ± 0.03	0.73 ± 0.02	<0.01
L	0.76 ± 0.04	0.71 ± 0.02	<0.0001
Posterior limb of internal capsule			
R	0.71 ± 0.03	0.67 ± 0.03	<0.0001
L	0.70 ± 0.04	0.67 ± 0.02	<0.01
Superior corona radiata			
R	0.74 ± 0.02	0.72 ± 0.02	<0.001
L	0.73 ± 0.03	0.71 ± 0.02	<0.01
Posterior thalamic radiation	0.82 ± 0.03	0.80 ± 0.03	<0.05
Sagittal stratum	0.82 ± 0.03	0.80 ± 0.03	<0.05
External capsule			
R	0.80 ± 0.02	0.78 ± 0.02	<0.05
L	0.79 ± 0.03	0.76 ± 0.02	<0.01
Fornix			
R	0.82 ± 0.03	0.78 ± 0.05	<0.01
L	0.80 ± 0.03	0.77 ± 0.02	<0.01
Superior fronto-occipital fasciculus	0.71 ± 0.04	0.69 ± 0.04	<0.05

### Relationship between FA and Clinical Variables

**Figure 3:**
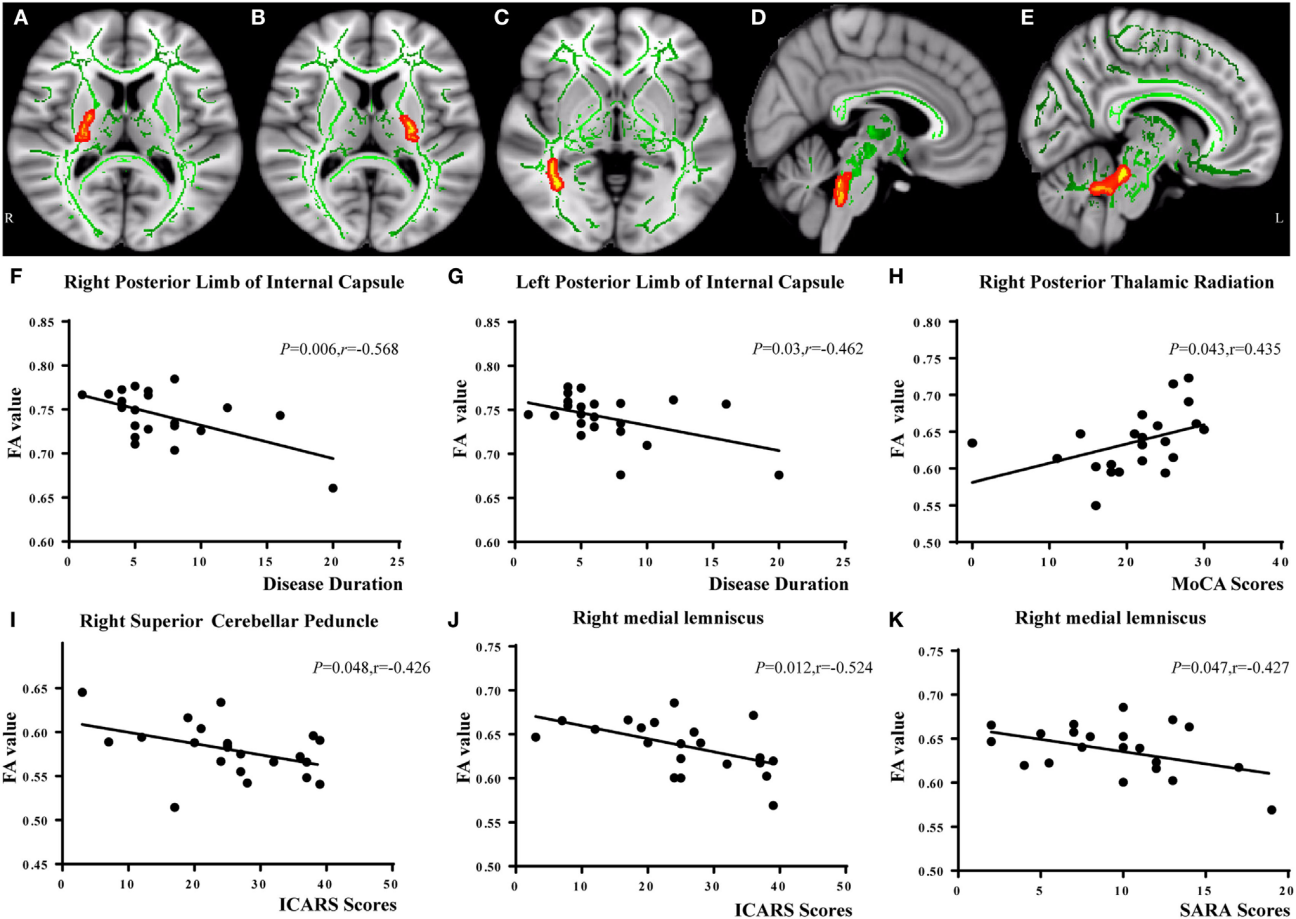
Correlation analysis between FA values and spinocerebellar ataxia type 3 disease severity. FA of bilateral posterior limb of internal capsule negatively correlated with disease duration [right (**A,F)**; left **(B,G)**]. FA of right posterior thalamic radiation **(C)** positively related with Montreal Cognitive Assessment (MoCA) scores **(H)**. FA of right superior cerebellar peduncle **(D)** was negatively correlated with International Cooperative Ataxia Rating Scale (ICARS) scores **(I)**. FA of right medial lemniscus **(E)** was negatively correlated with ICARS **(J)** and Scale for the Assessment and Rating of Ataxia (SARA) scores **(K)**.

In the correlation analysis, we found a moderately negative correlation between the mean FA value of the common WM skeleton and the ICARS scores of SCA3 patients (*r* = −0.535, *p* = 0.010, Figure 2C). For each subscale of the ICARS, we found inverse correlations between mean FA value and kinetic function (*r* = −0.538, *p* = 0.010, Figure 2D). Furthermore, the correlation analysis was conducted between the FA/MD value and clinical variables in SCA3 group, such as the onset age, disease duration, the ICARS, SARA, and MoCA scores. We found an inverse correlation between FA value of bilateral posterior limb of internal capsule and disease duration of SCA3 individuals (right: *r* = −0.568, *p* = 0.006, Figures 3A,F; left: *r* = 0.462, *p* = 0.03, Figures 3B,G). We also identified negative associations between FA values of the right SCP (*r* = −0.426, *p* = 0.048, Figures 3D,I), medial lemniscus (*r* = −0.524, *p* = 0.012, Figures 3E,J), and ICARS scores. A similar negative correlation was found between the right medial lemniscus (*r* = −0.524, *p* = 0.012, Figure 3K) and SARA scores. A positive correlation was found between FA value of the right posterior thalamic radiation (*r* = 0.435, *p* = 0.043, Figures 3C,H) and MoCA scores. In addition, we found ICARS positively correlated with MD in the MCP, left anterior limb of internal capsule, external capsule, and superior corona radiate (Table 4).

**Table 4:** White matter tracts showing correlation between mean diffusivity and International Cooperative Ataxia Rating Scale scores in spinocerebellar ataxia type 3.

	*r*	*p*-Value
Middle cerebellar peduncle	0.478	<0.05
Anterior limb of internal capsule L	0.445	<0.05
Superior corona radiata L	0.613	0.002
External capsule L	0.504	<0.05

## Discussion

In the present study, we used TBSS, an automated method, which circumvented the limitations caused by uncertainty of smoothing extent and by alignment inaccuracies, and found that WM tracts damage existed not only in SCA3 patients but also at an earlier disease stage, in asymptomatic mutation carriers.

### Partial WM Tracts Are Impaired in the Presymptomatic Stage of SCA3

In high-risk SCA3 patients, we found decreased FA values and increased MD in the left inferior and SCPs in presymptomatic SCA3 patients. The ICP mainly contains afferent fibers receiving information from movement centers, and a variety of sensory information related to movement (27). The SCP contains most of the efferent fibers projecting directly, or indirectly through the thalami, to the frontal cortex (28). These results indicate that microstructural changes of WM fibers in the cerebellum potentially exist before any detectable clinical manifestations in movement, during the asymptomatic stage of SCA3. This is in line with a previous study utilizing PET with fluorine-18-fluorodeoxyglucose (FDG), which found decreased FDG intake in the cerebellar hemispheres and brainstem of asymptomatic SCA3 gene carriers (29). In addition, our previous work using arterial spin labeling demonstrated that SCA3 mutation carriers showed a reduced cerebral blood flow in the cerebellar dentate nucleus (7, 30). In other types of SCA, Falcon and colleagues used fMRI and DTI during a passive smooth-pursuit task in 14 SCA6 patients and found activation in the vermis of the cerebellum in presymptomatic SCA6 individuals (31). Yoo and Oh (32) reported decreased FA values of cerebellar afferent and efferent pathways in a presymptomatic SCA1 patient compared to five age-matched healthy controls. These results suggest that abnormal WM tracts have existed in presymptomatic SCAs patients.

### WM Tract Abnormalities Are Widespread in SCA3

In the present study, SCA3 patients showed FA values of WM tracts significantly decreased across the whole brain, including both supratentorial and infratentorial regions. Increased MD was accordantly detected in supratentorial and infratentorial WM, which reflected widespread WM microstructural damage in SCA3. More specifically, the mean FA value of the mean FA skeleton showed a moderate inversed correlation with the ICARS scores of SCA3 patients. A negative association was also found between the mean FA value and the kinetic function subscale of the ICARS, which indicates that damage of WM tracts in the whole brain is related to ataxia severity in SCA3.

Previous studies mostly considered WM damage as less severe and important than GM abnormalities in the neuropathology of SCA3 (33). However, WM is crucial in connecting different brain regions and coordinating communication between them. WM studies have revealed abnormal WM in the cerebellum, brainstem, and spinal cord, including cerebellar WM, cerebellar peduncles, cranial nerves III–XII, medial and lateral lemniscus, vestibulospinal, medial longitudinal, spinocerebellar and spinothalamic tracts, cuneate and gracile fascicles in SCA3 patients (34–37). Horimoto (5) found MRI intensity changes in the internal capsule in SCA3, and autopsied cases have shown neurodegeneration of WM in the internal capsule. Using DWI, Guimaraes et al. (12) and Kang et al. (2) found FA reduction and MD increase in supratentorial WM in SCA3, which is consistent with our results. Research in other types of SCAs has demonstrated similar results. For example, decreased FA and increased MD in cerebellar peduncles, the bilateral posterior limb of the internal capsule and the corona radiate have been observed in SCA2 patients (38). WM FA reductions have also been found in the cerebellum, cerebellar, and cerebral peduncles, brainstem, anterior and posterior internal capsule, external/extreme capsule, corpus callosum, corona radiata, optical radiations, and the occipital, temporal, and frontal lobes in SCA7 patients (39). These findings indicate that there is widespread impairment in WM tracts in the whole brain across different SCA types, which is possibly due to similar pathological mechanisms.

FA describes the degree of anisotropy of water diffusion processes. It reflects fiber density, axonal diameter, intracellular organelles, cellular membranes, and myelination in WM (40). Evidence suggests that axonal loss may be the main reason for decreased FA in neural fiber tracts, although demyelination may be not necessary for significant anisotropy. Neuropathological studies have observed neuronal loss and degeneration in cerebellum, brainstem (41), and spinal cord, as well as thalamus (42), basal ganglia (5, 43), and the cerebral cortex (44), which would consecutively result in the axonal loss of neurons. This provides a potential explanation for the extensive WM involvement observed in our study.

### Specific WM Tracts Are Closely Related to SCA3 Disease Duration and Severity

In this study, we observed that FA values of bilateral posterior limb of internal capsule had an inverse relationship with disease duration of SCA3, indicating that lower FA values in posterior limb of internal capsule were associated with longer SCA3 disease duration. MD in left anterior limb of internal capsule and external capsule was positively associated with ICARS scores of SCA3 patients. There are a lot of sensory and motor fibers in the internal and external capsules, and they are crucial in movement dexterity and motor coordination (45). WM microstructural alterations in the internal and external capsules have been reported in SCAs (38, 39). However, no significant correlations were found between the alterations and SCA3 disease severity in previous studies. Our result is in line with a longitudinal MRI study in SCA3 patients (5), which found high intensity changes of the internal capsule in T2-weighted images and fluid-attenuated inversion recovery imaging, with changes showing progression as the disease progressed. Involvement of the posterior limb of the internal capsule may explain the more severe pyramidal signs observed in patients with longer disease duration.

Moreover, our result showed that FA values of the SCP and medial lemniscus were negatively related to SCA3 disease severity. Reduction of FA in the SCP has been reported in previous TBSS studies of SCA3 (6, 12) and other types of SCAs diseases (38, 46). These results are in accordance with SCA3 pathological studies that revealed neuron loss in the cerebellar dentate nucleus with myelin loss or atrophy of the SCP (33, 43). The medial lemniscus, connecting the brain stem and the thalamus, is associated with somatosensory dysfunctions such as decreased sense of vibration and kinesthesia, impairments of tactile and algesic sensation, and decreased position sense in SCA3 patients (47). Studies on individuals with SCA2 (39) and Friedreich Ataxia (48) have also demonstrated atrophy and microstructural damage in the medial lemniscus. A neuropathological study (49) has also shown neurodegeneration in the medial lemniscus in SCA3 patients with longer disease duration, which provides pathological evidence for our results. MD in the left superior corona radiate has shown a correlation with ICARS scores. The corona radiate, which projects to the entire cerebral cortex through the internal capsule, has been reported associated with ataxic hemiparesis (50). The similar results have been reported in SCA2 patients (39).

In our study, we also found significantly lower MoCA scores in SCA3 patients compared with healthy controls. Furthermore, lower FA value of the right posterior thalamic radiation in SCA3 patients was associated with lower MoCA scores, indicating that patients with lower FA value in right posterior thalamic radiation were with comparatively more serious cognitive dysfunctions (51, 52). Cognitive impairment in SCA3 patients can be explained by either cerebellar and extracerebellar pathology, or disruption of cerebellar-cerebral circuitries (53). The posterior thalamic radiation is also related to cognition in some way. Previous researchers have revealed that posterior thalamic radiation damage was related to cognition in essential tremor (54) and may also be associated with intellectual performance (55).

### Limitations and Future Directions

The present study found impaired WM tracts in SCA3 patients and mutation carriers with no clinical symptoms, compared with NC. However, some limitations of this research must be considered. First, our sample size is relatively small. However, we set stringent thresholds for statistical significance, and differences observed between SCA3 and NC were still strongly significant, supporting the reliability of the data. Second, the present study is cross-sectional. Future studies using longitudinal imaging data to investigate microstructural changes in SCA3 patients are necessary to corroborate these findings. Long-term follow-up data will also be necessary to evaluate the usefulness of MRI as a potential marker of disease progression in SCA3.

## Conclusion

White matter tracts are impaired even in the asymptomatic stage of SCA3. Abnormality of WM fibers in SCA3 patients is widespread and severe, compared with NC. Specific WM tracts are closely related to SCA3 disease severity, including both movement disorders and cognitive dysfunctions. These results can deepen our understanding of the neural basis of SCA3 dysfunction.

## Ethics Statement

All procedures performed in studies involving human participants were approved by the Ethics Committee of Xiangya Hospital, Central South University in China, which was in accordance with the ethical standards of the institutional and/or national research committee and with the 1964 Helsinki declaration and its later amendments or comparable ethical standards. And written informed consent was obtained from all subjects.

## Author Contributions

XW: study conception, design, and organization, acquisition of data, analysis and interpretation of data, drafting of the manuscript, critical revision of the manuscript for important intellectual content, statistical analysis, administrative, technical, and material support, study supervision. XL: study conception, design, and organization, acquisition of data. YZ: study conception, design, and organization, analysis and interpretation of data, statistical analysis. CC: statistical analysis, administrative, technical, and material support, study supervision. WS: study conception, design, and organization, acquisition of data. MH: acquisition of data. ZZ: acquisition of data, analysis and interpretation of data, statistical analysis. ZW: analysis and interpretation of data, statistical analysis. ZQ: analysis and interpretation of data, critical revision of the manuscript for important intellectual content, statistical analysis. WX: acquisition of data. WL: acquisition of data, analysis and interpretation of data, statistical analysis, administrative, technical, and material support. BT: study conception, design, and organization, analysis and interpretation of data, drafting of the manuscript, critical revision of the manuscript for important intellectual content, statistical analysis, administrative, technical, and material support, study supervision. LS: study conception, design, and organization, analysis and interpretation of data, drafting of the manuscript, critical revision of the manuscript for important intellectual content, statistical analysis, administrative, technical, and material support, study supervision.

## Conflict of Interest Statement

The authors declare that the research was conducted in the absence of any commercial or financial relationships that could be construed as a potential conflict of interest.
