# Supplementary material for: Microstructural Alterations in Asymptomatic and Symptomatic Patients with Spinocerebellar Ataxia Type 3: A Tract-Based Spatial Statistics Study
Source: Front Neurol. 2017 Dec 22;8:714. doi: 10.3389/fneur.2017.00714 (PMC5744430; doi:10.3389/fneur.2017.00714)
Supplement: Supplementary file 1 [file Table_1.docx]

**STable 1**: Demographics of participants in this study

|  | | **preSCA3**  **(n=16)** | **Control for preSCA3**  **(n=20)** | **SCA3**  **(n=22)** | **Control for SCA3**  **(n=18)** | ***p* value** |
| --- | --- | --- | --- | --- | --- | --- |
| Age (years) | | 28.81±7.19 | 32.05±9.26 | 43.36±5.89 | 39.22±11.42 | 0.416 preSCA3 vs control  0.121 SCA3 vs control  <0.01 pre-SCA3 vs SCA3 |
| Disease duration (years) | | - | - | 6.95±4.36 | - | - |
| Gender ( male/female) | | 5/11 | 10/10 | 14/8 | 8/10 | 0.257 preSCA3 vs control  0.225 SCA3 vs control |
| MoCA | | 27.69±1.62 | 26.75±2.53 | 21.0±6.9 | 25.78±2.82 | <0.01 preSCA3 vs SCA3 |
| SARA | | 0.25±0.45 | - | 9.5±4.39 | - | <0.01 preSCA3 vs SCA3 |
| ICARS | Total score  Posture and gait disturbance  Limb kinetic functions  Speech disturbance  Oculomotor disorders | 1.12±0.88  2.25±1.25 | - | 25.55±10.12  10.82±5.36  9.95±7.36  2.55±1.82 | - | <0.01 preSCA3 vs SCA3 |

*MoCA: Montreal cognitive assessment. SARA: Score of Scale for the Assessment and Rating of Ataxia. ICARS: International Cooperative Ataxia Rating Scale*

*Data were presented mean ± standard deviation (SD).*
